# Administration of nimotuzumab combined with cisplatin plus 5-fluorouracil as induction therapy improves treatment response and tolerance in patients with locally advanced nasopharyngeal carcinoma receiving concurrent radiochemotherapy: a multicenter randomized controlled study

**DOI:** 10.1186/s12885-019-6459-6

**Published:** 2019-12-30

**Authors:** Ying Lu, Dagui Chen, Jinhui Liang, Jianquan Gao, Zhanxiong Luo, Rensheng Wang, Wenqi Liu, Changjie Huang, Xuejian Ning, Meilian Liu, Haixin Huang

**Affiliations:** 1 Department of Oncology, the Fourth Affiliated Hospital of Guangxi Medical University, Liuzhou, China; 2 Department of Radiotherapy, Wuzhou Red Cross Hospital, Wuzhou, China; 3 Department of Radiotherapy, Liuzhou People’s Hospital, Liuzhou, China; 4 Department of Radiotherapy, the First Affiliated Hospital of Guangxi Medical University, Nanning, China; 5 Department of Radiotherapy, the Second Affiliated Hospital of Guangxi Medical University, Nanning, China; 6 Department of Oncology, the Second People’s Hospital of Nanning, Nanning, China; 7 Department of Oncology, Liuzhou Traditional Chinese Medical Hospital, Liuzhou, China; 8 Department of Radiotherapy, the Affiliated Hospital of Guilin Medical College, Guilin, China

**Keywords:** Locally advanced nasopharyngeal carcinoma, EGFR monoclonal antibody, Induction therapy, Curative effect, Adverse reaction

## Abstract

**Background:**

Nimotuzumab (NTZ) is an anti-EGFR monoclonal antibody. However,the effect of targeted drugs combined with induction therapy in locally advanced nasopharyngeal carcinoma remains unclear. The aim of this study is to investigate the safety and efficacy of NTZ combined with cisplatin plus 5-fluorouracil (PF) as induction regimen in locally advanced nasopharyngeal carcinoma (NPC) patients receiving concurrent radiochemotherapy.

**Methods:**

This was a multicenter randomized controlled study performed in eight Guangxi hospitals in 2015–2017. Eligible patients with NPC were randomized into nimotuzumab/PF (NPF group) and docetaxel/PF (DPF group) regimens, respectively, as induction therapy. After 2 cycles of induction therapy, all patients received cisplatin and concurrent intensity modulated radiation therapy (IMRT). Then, the two groups were compared for safety and efficacy.

**Results:**

A total of 118 patients with stage III-IVa NPC were assessed, with 58 and 60 in the NPF and DPF groups, respectively. Compared with DPF treatment, NPF induction therapy showed a more pronounced effect on cervical lymph nodes (*P* = 0.036), with higher response rate (RR) (81% vs 60%). Compared with the DPF group, the NPF group showed significantly reduced leukopenia, neutropenia and gastrointestinal reactions (all *P* < 0.05); rash only appeared in the NPF group, but all cases were grade 1. During concurrent treatment with radiotherapy and chemotherapy, the NPF group showed better tolerance to radiotherapy and chemotherapy; neutropenia, anemia, gastrointestinal reactions, oral mucositis and radiation dermatitis in the NPF group were significantly reduced (*P* < 0.05). The expression rate of EGFR was 94.9% (112/118). Compared with the DPF group, patients with EGFR expression in the NPF group showed better response (77.8% vs 63.0%, *P* = 0.033).

**Conclusion:**

For locally advanced NPC patients receiving follow-up cisplatin and IMRT, nimotuzumab/PF for induction therapy has better lymph node response rate and milder adverse reactions than the DPF regimen. In addition, the patients have better tolerance in subsequent concurrent radiotherapy and chemotherapy; however, long-term efficacy needs further follow-up evaluation.

**Trial registration:**

The registration number of the clinical trial is ChiCTR-OIC-16008201 and retrospectively registered on March 31, 2016.

## Background

Nasopharyngeal carcinoma (NPC) is a head and neck cancer with a unique biological behavior; unlike other head and neck tumors, NPC has higher sensitivity to radiotherapy and chemotherapy [1–3]. Therefore, radiochemotherapy is the main treatment option for locally advanced NPC. With the application of intensity modulated radiation therapy (IMRT), distant metastasis has become a major factor affecting prognosis in NPC [3]. Induction therapy may reduce the micrometastasis of NPC, better radiotherapeutic conditions for locally advanced nasopharyngeal carcinoma (especially in patients with giant lesions), and improve patient survival and prognosis [4]. Based on concurrent radiotherapy and chemotherapy (CCRT), induction chemotherapy (IC) could increase the 5-year absolute benefit rates of progression-free survival (PFS) and distance control (DC) by 4.2 and 8.7%, respectively reduce the risk of cancer-related death by 4.8% [5], and improve the overall survival (OS) rate of patients [6]. IC-CCRT is the most effective DC regimen (HR = 0.44, 95%CI 0.27–0.71; P-score 95%) in the comprehensive treatment of NPC. It improves OS (HR = 0.81, 95%CI 0.63–1.04; P-score 63%) and PFS (HR = 0.68, 95%CI 0.54–0.85; P-score 95%) [5]. Therefore, induction chemotherapy combined with concurrent radiochemotherapy is considered an effective therapeutic mode for locally advanced NPC [4, 7], but the optimal induction therapy remains uncertain.

The DPF regimen consisting of docetaxel, cisplatin and 5-fluorouracil shows a higher objective remission rate (ORR) compared with the cisplatin plus 5-fluorouracil (PF) regimen. It is recommended by category I of NCCN guidelines for diagnosis and treatment of head and neck squamous cell carcinoma [8, 9], Induction therapy with the DPF regimen is also used clinically for NPC patients, in whom the ORRs of primary lesions and cervical lymph nodes after DPF induction chemotherapy could reach 92.9% [10]. Based on concurrent radiochemotherapy, DPF chemotherapy effectively improves 3-year OS (86% vs 92%, *P* = 0.029) and distant metastasis-free survival (DMFS) (83% vs 90%, *P* = 0.031) [11]. In N2–3 NPC patients, the 3-year distant metastasis rate decreases by 26% (*P* = 0.08) while OS increases by 25% (*P* = 0.21) [12]. Compared with induction chemotherapy such as administration of docetaxel combined with cisplatin (TP) or PF, the DPF regimen alone significantly increases PFS (HR = 0.70; 95%CI 0.49–0.95), OS (HR = 0.59; 95%CI 0.37–0.92) [6]. However, while the DPF regimen achieves higher efficacy, treatment-related adverse reactions also increase significantly, especially the incidence of grade 3–4 neutropenia whose rate could reach 42% [11, 13], which hampers the clinical application of DPF.

With the gradual development of molecular biology research, molecular targeted therapy has become a research hotspot in cancer therapy. The expression rate of epidermal growth factor receptor (EGFR) in nasopharyngeal carcinoma is 68–89%, which is much higher than that of other solid tumors [14]. Meanwhile, EGFR expression is closely related to prognosis in NPC [15–17]. Overexpression of EGRF significantly increases the risk of adverse prognosis of NPC OS (HR = 1.86, 95%CI 1.25–2.77; *P* = 0.000),disease-free survival (DFS) (HR = 2.25, 95%CI 1.66–3.04; *P* = 0.000), locoregional recurrence free survival (LRFS) (HR = 2.93, 95%CI 1.71–5.02; *P* = 0.000) [15]. In patients with locally advanced NPC receiving IMRT, combined anti-EGFR receptor therapy may be a more effective treatment strategy [18].

Nimotuzumab (NTZ) is a humanized monoclonal antibody [19, 20]. Compared with cetuximab (CTX), NTZ is human-derived and highly selective, with a long half-life. It competitively inhibits the binding of endogenous ligands to EGFR and blocks the downstream signal transduction pathway mediated by EGFR, thereby inhibiting the proliferation of tumor cells, promoting apoptosis of tumor cells, suppressing angiogenesis and increasing radiosensitivity to chemotherapy; meanwhile, it has few adverse reactions and low incidence of rash. Therefore, whether combined with CCRT or applied as an induction therapeutic, skin rash and mucosal reactions of nimotuzumab are significantly improved compared with those of cetuximab. Retrospective analysis suggested that sequential concurrent radiochemotherapy after induction chemotherapy combined with NTZ is effective and well tolerated in the treatment of locally advanced NPC [21]. In addition, it was demonstrated that NTZ combined with concurrent chemoradiotherapy is beneficial in the treatment of locally advanced NPC, with limited toxicity and good-tolerability [22]. However, there is a lack of prospective analysis of nimotuzumab for induction therapy in locally advanced NPC. Therefore, the current randomized controlled study aimed to assess the safety and efficacy of NTZ combined with PF as induction regimen in locally advanced NPC patients receiving concurrent radiochemotherapy.

## Methods

### Patients

This randomized controlled study assessed NPC patients receiving initial treatment in eight hospitals in Guangxi (Oncology Department of the Fourth Affiliated Hospital of Guangxi Medical University, Radiotherapy Department of Wuzhou Red Cross Hospital, Radiotherapy Department of Liuzhou People’s Hospital, Radiotherapy Department of the First Affiliated Hospital of Guangxi Medical University, Radiotherapy Department of the Second Affiliated Hospital of Guangxi Medical University, Oncology Department of Nanning Second People’s Hospital, Oncology Department of Liuzhou Traditional Chinese Medicine Hospital, Radiotherapy Department of the affiliated hospital of Guilin Medical College) from January 2015 to December 2017.

Inclusion criteria were: 1) 18–70 years old; 2) undifferentiated non-keratinizing nasopharyngeal carcinoma pathologically diagnosed by biopsy; 3) clinical stage III-IVa (08 Chinese stage); 4) KPS score ≥ 70; 5) serum hemoglobin ≥10 mg/dL, platelet ≥10,000/mu L, and absolute neutrophil count ≥1500/mu L; 6) serum creatinine ≤1.5 times UNL or creatinine clearance rate ≥ 60 ml/min; bilirubin ≤1.5 times UNL and AST (SGOT) and ALT (SGPT) ≤ 1.5 times UNL; 8) estimated survival time ≥ 6 months.

Exclusion criteria were: 1) previous diagnosis of malignant tumors; 2) previous radiotherapy, chemotherapy, or targeted therapy; 3) radiotherapy or chemotherapy contraindications; 4) allergy to any study drug.

The study was approved by the Ethics Committee of the Fourth Affiliated Hospital of Guangxi Medical University (PJK2015201). The registration number of the clinical trial is ChiCTR-OIC-16008201, and all patients provided signed informed consent obtained was written.

### Treatments

In this open-label trial, allocation concealment was performed before eligible patients were randomly divided into two groups: NPF (induction therapy with nimotuzumab combined with PF; nimotuzumab 200 mg/time/week by intravenous drip, cisplatin 75 g/m^2^ by intravenous drip at d1, and continuous infusion of 5-fluorouracil 750 mg/m^2^/day at d1–5; repeated every 3 weeks) and DPF (induction chemotherapy; docetaxel 75 g/m^2^ by intravenous drip at d1, cisplatin 75 g/m^2^ by intravenous drip at d1, and continuous infusion of 5-fluorouracil 750 mg/m^2^/d at d1–5; repeated every 3 weeks) groups. After 2 cycles of induction therapy, cisplatin (80 g/m^2^ by intravenous drip at d1, repeated every 3 weeks, for a total of 3 cycles) was administered to both groups concurrently with intensity modulated radiation therapy (IMRT). G-CSF was not administered prophylactically before the first induction therapy or concurrent chemotherapy; in patients with grade 4 neutropenia, prophylactic G-CSF leucocyte-raising therapy was used during the follow-up cycle. In patients who still showed intolerability, chemotherapeutic drug dosage was reduced by 15% according to the principle of reduction of chemotherapeutic drugs. All chemotherapy cycles routinely included antiemetic treatment, and the follow-up cycle antiemetic regimen was adjusted according to gastrointestinal reactions.

All patients were treated by IMRT. The range of tumors was determined by magnetic resonance (MR), and the target area was delineated and planned by enhanced CT. According to the actual situation of each center, the planning target volume (PTV) formed by expanding 3–5 mm from each target area was administered a prescription dose: nasopharyngeal tumor volume (GTVnx) and cervical lymph node volume (GTVnd) at 69.69–70.06 Gy and 64.17–70.06 Gy, respectively; high-risk area of primary focus (CTV1) at 66.03 Gy; low-risk area of primary focus and cervical lymph node drainage area (CTV2) at 50.4–54.25 Gy. The number of segmentations was 31–33 times, 5 times a week. According to the requirements of RTOG 0615 and RTOG 0225, organ-threatening dose and planning were assessed.

### Evaluation indexes

The primary endpoints were the efficacy of induction therapy and adverse reactions to induction therapy. The secondary endpoints were immediate and 3-month follow-up effects of the whole therapy and adverse reactions to concurrent chemoradiotherapy. The acute and late side effects of radiotherapy were evaluated according to RTOG acute and late radiation reaction scoring criteria, and the adverse reactions related to chemotherapy were assessed according to NCI-CTC AE3.0 classification criteria of common adverse drug reactions. The curative effects were based on Response Evaluation Criteria in Solid Tumors (RECIST 1.1) [23] and included complete response (CR; no detectable cancer after your treatment), partial response (PR; at least 30% decrease in total lesion area), stable disease (SD; no change or decrease of total lesion area below 30%) and progressive disease (PD; increase in total lesion area).

### EGFR detection

EGFR expression in nasopharyngeal carcinoma tissues was evaluated by immunohistochemical staining. The tumor tissues were fixed with 10% formaldehyde, paraffin embedded and sliced into 4-μm sections. After conventional dewaxing, endogenous peroxidase activity was blocked by treatment with hydrogen peroxide, followed by antigen retrieval via heating. Then, the samples were successively incubated with primary (overnight at 4 °C) and biotinylated secondary (37 °C for 30 min) antibodies, and treated with DAB for 10 min. After dehydration with graded ethanol, counterstaining was performed with hematoxylin. The range and intensity of staining were observed and recorded under a microscope. Phosphate buffer saline was used as a negative control, and known positive samples were used as positive controls. Grading was performed according to staining intensity and the percentage of positive cells. Positive signals were dark blue-purple granules in the cell membrane or cytoplasm of the tumor cells; positive cells were grouped into 0–4%, 5–24%, 25–49%, 50–75 and > 75%. Two deputy directors of pathology interpreted the results independently.

### Statistical methods

In the current non-inferiority study, the efficacy of induction therapy was the primary point for non-inferiority and RR decreases less than 5% as non-inferiority margin. We used an alpha of 0.05 and a beta of 0.20. Considering a dropout rate of 10%, we found that 60 was an appropriate sample size for each group. Therefore, a total of 120 patients were enrolled. IBM SPSS 19.0 was used for statistical analysis. Measurement data were analyzed using t test, while count data were compared by the χ2 test. Differences were considered significant at *p* < 0.05.

## Results

### Baseline patient characteristics

**Fig. 1:**
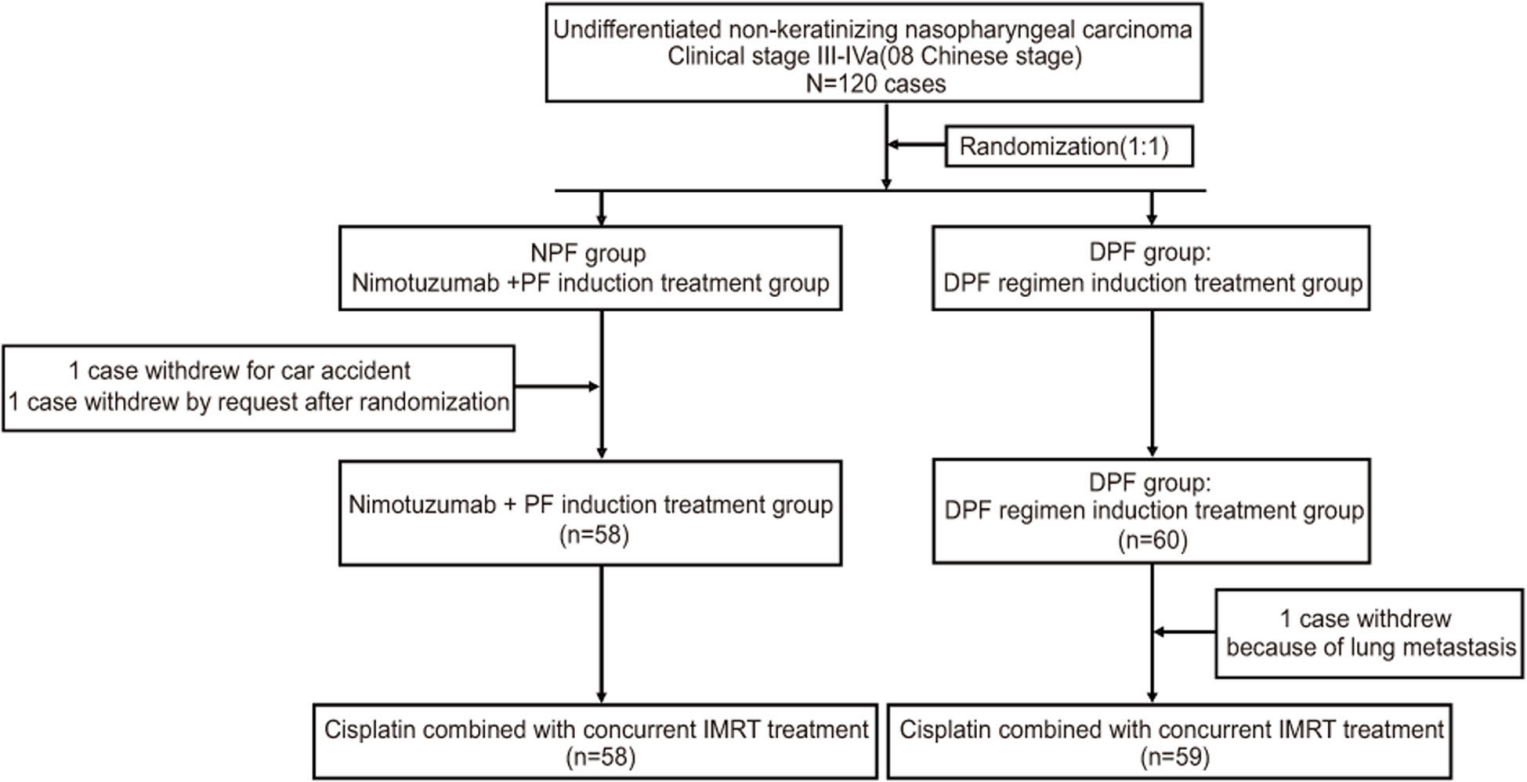
Study flowchart

From May 2015 to November 2017, a total of 118 patients were enrolled in the current study. The study flowchart was shown in Fig. 1. The average age of all patients was 44 years (22–68 years). There were 98 males and 20 females, indicating a male to female ratio of 4.9. A total of 66 cases had stage III disease while 52 had stage IVa. The NPF and DPF groups comprised 58 and 60 cases, respectively. There were no significant differences in baseline characteristics, including age, sex, and clinical stage between the two groups (*P* > 0.05) (Table 1).

**Table 1:** Clinical characteristics of the two groups

Characteristics	No. of patients (%)			P
	Total (*n* = 118)	NPF group (*n* = 58)	DPF group (*n* = 60)	
Median age (range)	44(22–68)	43(22–65)	45(22–68)	0.214
Gender				
Males	98(83.1)	47(81.0)	51(85.0)	0.371
Females	20(16.9)	11(19.0)	9(15.0)	
Clinical stage (China, 2008)				
III	66(55.9)	34(58.6)	32(53.3)	0.347
IVa	52(44.1)	24(41.4)	28(46.7)	
T stage				0.436
T1	6(5.1)	3(5.1)	3(5.0)	
T2	20(16.9)	12(20.7)	8(13.3)	
T3	57(48.3)	27(46.6)	30(50.0)	
T4	35(29.7)	16(27.6)	19(31.7)	
N stage				0.585
N0	3(25.4)	2(3.4)	1(1.6)	
N1	27(22.9)	15(25.9)	12(20.0)	
N2	67(56.9)	30(51.7)	37(61.7)	
N3	21(17.8)	11(19.0)	10(16.7)	

### Therapeutic effects

By September 2018, all patients had completed induction therapy and therapeutic effects were evaluated. In the induction stage, compared with the DPF group, NPF induction therapy had more pronounced effects on cervical lymph nodes (*P* = 0.036) and RR (CR + PR) (81% vs 60%). There were no significant differences in nasopharyngeal lesions and overall efficacy (*P* = 0.446, *P* = 0.143) between the two groups. One case of lung metastasis after induction chemotherapy in the DPF group exited the study. A total of 117 patients were further treated with cisplatin and concurrent IMRT, including 58 and 59 in the NPF and DPF groups, respectively. There were no significant differences between the two groups (*P* = 0.449 and *P* = 0.409) in immediate efficacy evaluation and 3-month efficacy evaluation at the end of the whole course of treatment (Table 2).

**Table 2:** Therapeutic effects in the two groups

Therapeutic effect^a^	After induction therapy (*n* = 118)						After concurrent radiochemotherapy (*n* = 117)^b^		3 months after the treatment (*n* = 117)	
	Nasopharyngeal lesions (%)		Lymph node lesions (%)		Total(%)(nasopharyngeal and lymph node lesions)					
	NPF group (*n* = 58)	DPF group (*n* = 60)	NPF group (*n* = 58)	DPF group (*n* = 60)	NPF group (*n* = 58)	DPF group (*n* = 60)	NPF group (*n* = 58)	DPF group (*n* = 59)	NPF group (*n* = 58)	DPF group (*n* = 59)
CR	2(3.4)	0(0)	5(8.6)	1(1.7)	2(3.4)	0(0)	39(67.2)	38(64.4)	51(87.9)	50(84.7)
PR	33(56.9)	31(51.7)	42(72.4)	35(58.3)	39(67.2)	37(61.7)	19(32.8)	21(35.6)	7(12.1)	9(15.3)
SD	23(39.7)	29(48.3)	11(19.0)	24(40.0)	17(29.3)	22(36.7)	0(0)	0(0)	0(0)	0(0)
PD	0(0)	0(0)	0(0)	0(0)	0(0)	1(1.7)	0(0)	0(0)	0(0)	0(0)
P	0.446		0.036		0.143		0.499		0.409	

^a^*CR* Complete remission, *PR* Partial remission, *SD* Stable disease, *PD* Progressive disease

^b^ After induction therapy, 1 patient in the DPF group showed distant metastasis and withdrew from the study

### Adverse reactions

In the induction stage, the main adverse reactions in the two groups were grade 1–2 leukopenia, neutropenia and gastrointestinal reactions. Compared with the DPF group, the NPF group showed significantly reduced leucopenia, neutropenia and gastrointestinal reactions (*P* = 0.037, *P* = 0.018 and *P* = 0.032, respectively). Rashes only appeared in the NPF group, and all were grade 1; after NTZ treatment, rashes could disappear spontaneously. There were no significant differences in hemoglobin decrease, thrombocytopenia, liver or kidney impairment, and oral mucositis (*P* > 0.05). In the concurrent radiotherapy and chemotherapy phase, the NTP group showed better treatment tolerance. Neutropenia, anemia, gastrointestinal reactions, oral mucositis and radiation dermatitis were significantly reduced in the NTP group compared with DPF group (*P* = 0.033, *P* = 0.049, *P* = 0.037, *P* = 0.020 and *P* = 0.035, respectively). Leukopenia, thrombocytopenia, and liver and kidney functions were also improved, but the differences were not statistically significant (*P* > 0.05) (Table 3).

**Table 3:** Adverse reactions in the two groups

Adverse reaction	Induction therapy stage (*n* = 118) (%)			Concurrent radiochemotherapy stage (*n* = 117) (%)		
	NPF group (*n* = 58)	DPF group (*n* = 60)	*P*	NPF group (*n* = 58)	DPF group (*n* = 59)	*P*
Leukocytopenia			0.037			0.090
0	20(34.5)	17(28.3)		14(24.1)	11(18.6)	
1	28(48.3)	21(35.0)		29(50.0)	24(40.7)	
2	7(12.1)	13(21.7)		14(24.1)	20(33.9)	
3	3(5.2)	8(13.3)		1(1.7)	4(6.8)	
4	0(0)	1(1.7)		0(0)	0(0)	
Neutropenia			0.018			0.033
0	19(32.8)	17(28.3)		13(22.4)	9(15.3)	
1	27(46.6)	19(31.7)		27(46.6)	20(37.3)	
2	9(15.5)	11(18.3)		14(24.1)	22(37.3)	
3	3(5.2)	9(15.0)		4(6.9)	6(10.2)	
4	0(0)	4(6.7)		0(0)	2(3.4)	
Anemia			0.247			0.049
0	49(84.5)	47(78.3)		42(72.4)	33(56.9)	
1	9(15.5)	11(18.3)		16(27.6)	23(39.7)	
2	0(0)	2(3.3)		0(0)	2(3.4)	
Thrombocytopenia			0.452			0.532
0	53(91.4)	53(88.3)		55(94.8)	55(93.2)	
1	5(8.6)	6(10.0)		3(5.2)	3(5.1)	
2	0(0)	1(0.8)		0(0)	1(1.7)	
Liver function damage			0.275			0.178
0	49(84.5)	43(71.7)		46(79.3)	41(69.5)	
1	7(12.1)	15(21.6)		12(20.7)	17(28.8)	
2	1(1.7)	2(3.3)		0(0)	1(1.7)	
3	1(1.7)	0(0)		0(0)	0(0)	
Renal function damage			0.166			0.254
0	55(94.8)	53(88.3)		55(94.8)	53(88.3)	
1	3(5.2)	6(10.0)		3(5.2)	6(10.0)	
2	0(0)	1(1.7)		0(0)	0(0)	
Gastrointestinal reaction			0.032			0.037
0	8(13.8)	3(5.0)		9(15.5)	4(6.8)	
1	38(65.5)	37(61.7)		32(55.2)	28(47.5)	
2	11(19.0)	16(26.7)		16(27.6)	25(42.4)	
3	1(1.7)	4(6.7)		1(1.7)	2(3.4)	
Oral mucositis			0.099			0.020
0	55(94.8)	51(85.0)		12(20.7)	6(10.2)	
1	2(3.4)	6(10.0)		27(46.6)	23(39.0)	
2	1(1.7)	3(5.0)		19(32.8)	28(47.5)	
3	0(0)	0(0)		0(0)	2(3.4)	
skin rash			0.012			
0	52(89.7)	60(100)		–	–	–
1	6(10.3)	0(0)		–	–	–
Radiation skin reaction						0.035
0	–	–		8(13.8)	6(10.2)	
1	–	–		34(58.6)	25(42.4)	
2	–			16(27.6)	26(44.1)	
3	–	–		0(0)	2(3.4)	

### Association of EGFR expression with the efficacy of induction therapy

The overall expression rate of EGFR was 94.9% (112/118), including 94.8% (55/58) and 95.5% (57/60) in the NPF and DPF groups, respectively. There was no significant difference in EGFR expression levels between the two groups (*P* = 0.058) (Table 4). EGFR expression was not significantly correlated with the efficacy of induction chemotherapy with DPF (*P* = 0.090), but significantly affected the efficacy of induction therapy combined with nimotuzumab (*P* = 0.015); compared with chemotherapy, induction therapy combined with nimotuzumab had better response (77.8% vs 63.0%,*P* = 0.033)., as shown in Table 5.

**Table 4:** Expression of EGFR in both patient groups

EGFR expression	NPF group (%)	DPF group (%)	Total (%)
0–4%	3(5.2)	3(5.0)	6(5.1)
5–24%	10(17.2)	3(5.0)	13(17.2)
25–49%	9(15.5)	8(13.3)	17(14.4)
50–74%	26(44.8)	29(48.3)	55(46.6)
75–100%	10(17.2)	17(28.3)	27(22.9)
P	0.058

**Table 5:** Correlation between EGFR expression and the curative effect of induction therapy

EGFR expression	NPF group (*n* = 58)				DPF group (*n* = 60)				
	CR	PR	SD	PD	CR	PR	SD	PD	P
0–4%	0	1	1	0	0	3	0	0	
5–24%	0	4	6	0	0	1	2	0	
25–49%	1	7	1	0	0	4	4	0	0.033
50–74%	0	18	8	0	0	23	6	1	
75–100%	1	9	1	0	0	6	10	0	
P	0.015				0.09				\

## Discussion

This study assessed the effectiveness of NTZ combined with PF as induction regimen in locally advanced NPC cases receiving concurrent radiochemotherapy, and demonstrated that nimotuzumab combined with PF for induction therapy has better lymph node response rate and milder adverse reactions compared with the DPF regimen. In addition, the patients showed improved tolerance in subsequent concurrent radiotherapy and chemotherapy.

In a study by Chua DT [24] EGFR was shown to be expressed in 89% of nasopharyngeal carcinoma cases, and high EGFR expression is considered an independent prognostic factor for local control, non-recurrence and disease-related survival in stage III-IV NPC. In the latter report, 72% of patients with EGFR expression (> 25%) showed significant adverse prognosis after induction chemotherapy and radiotherapy. It was therefore suggested that anti-EGFR therapy might be necessary to improve prognosis in locally advanced NPC with high EGFR expression to increase clinical benefits. In the present study, 94.9% of NPC patients expressed EGFR, including 77.7% whose EGFR expression exceeded 25%, corroborating Chua’s study [24] The efficacy of induction therapy combined with anti-EGFR was related to EGFR (*P* = 0.015). Meanwhile, supplementing anti-EGFR monoclonal antibody significantly affected the efficacy of induction therapy (*P* = 0.033), suggesting that induction therapy combined with anti-EGFR therapy is feasible.

One of the aims of induction therapy is to effectively alleviate the lesions and create improved radiotherapy conditions for NPC, especially in patients with giant lesions, achieving better prognosis. For instance, in NPC patients undergoing follow-up CCRT, 5-year OS rates in the CR, PR and SD subgroups after induction chemotherapy were shown to be 100, 79.4 and 60%, respectively. The efficacy of induction therapy may therefore affect patient survival and prognosis [25]. It was reported that induction therapy with taxanes significantly increases ORR (OR = 4.57, 95%CI 1.14–18.30, *P* = 0.032, z = 2.15) [26] compared with the non-taxane regimen. However, in this study, induction therapy with NPF had higher RR in lymph node lesions (81.0% vs 60%) compared with DPF. These findings suggest that induction therapy combined with EGFR is more effective in alleviating lesions, creating better radiotherapy conditions and improving survival and prognosis in NPC with high EGFR expression.

With the application of IMRT, distant metastasis has become a major factor affecting prognosis in NPC. Meanwhile, induction therapy may reduce the micrometastasis of locally advanced NPC, and is considered an effective control scheme for distant metastasis in various comprehensive treatment regimens, improving PFS and OS [5]. Based on concurrent radiochemotherapy, the DPF regimen was added to induce chemotherapy, which effectively increased the 3-year OS (*P* = 0.029) and DMFS (*P* = 0.031) [11]. Meanwhile, the 3-year distant metastasis rate of NPC patients with N2–3 disease decreased by 26% (*P* = 0.08), while OS increased by 25% (*P* = 0.21) [12]. Among the various induction therapies commonly used in clinic, the PF regimen significantly reduces the risk of adverse prognosis of PFS (HR = 0.75; 95%CI, 0.56–0.99), whereas the DPF regimen increases the risk of adverse prognosis of OS while improving PFS (HR = 0.59; 95%CI, 0.37–0.92) [6]. However, adding EGFR monoclonal antibody based on DPF does not significantly increase the survival advantage of NPC patients. NTZ combined with DPF does not improve 5-year OS (89.9% vs 93.3%) and PFS (79.3% vs 82.1%) compared with NTZ combined with PF [21]. Therefore, EGFR combined with PF may represent a more economical and effective induction therapeutic regimen for NPC. As shown above, there was no significant difference between the NPF and DPF groups during immediate and 3-month curative effect evaluation. Although long-term survival data for NPF induction therapy and DPF induction chemotherapy were not available in this study, N stage is the main prognostic factor of DMFS and OS in nasopharyngeal carcinoma. A higher regional lymph node response rate (*P* = 0.036) after NPF induction therapy would help control regional lymph node-related distant metastasis and improve survival. It is worth assessing long-term survival after treatment with the NPF regimen.

On the other hand, induction therapy by NTZ combined with PF reduces the intensity of chemotherapy compared with DPF, which is helpful in improving hematological toxicity that restricts the clinical application of DPF. Adverse reactions caused by induction chemotherapy, especially increased systemic toxicity, hamper the application of induction chemotherapy and follow-up concurrent radiochemotherapy to a certain extent. It was reported that the incidence of grade 3–4 neutropenia in taxane-based induction therapy is significantly higher than that of non-taxane containing regimens (39.1% vs 16.1%, OR = 3.62, 95%CI 2.42–5.40, *P* < 0.001, z = 6.29) [26]. The incidence rates of neutropenia and stomatitis were 42 and 41%, respectively, after addition of the DPF regimen based on CCRT, which affected compliance with IC and subsequent CCRT. Adding anti-EGFR therapy to standard therapy (radiotherapy or radiochemotherapy) significantly increases OS (HR = 0.51; 95%CI, 0.39–0.66) and DFS (HR = 0.68; 95%CI, 0.54–0.86); meanwhile, systemic toxicity significantly decreases when EGFR therapy replaces cytotoxic drugs at the time of radiotherapy [27]. In NPC patients administered IMRT, CTX/NTZ combined with induction therapy can reduce severe toxicity and achieve better survival benefit compared with CTX/NTZ applied in concurrent radiotherapy [28]. These findings suggest that anti-EGFR monoclonal antibody combined with an appropriate intensity of chemotherapy may constitute an effective induction therapy to ensure or increase efficacy while markedly alleviating toxic reactions. Because of humanization, NTZ has better tolerance in nasopharyngeal carcinoma treatment [21, 29]. Compared with CTX, NTZ significantly improves skin rash (*P* < 0.001), oral mucositis (*P* < 0.001) and weight loss (*P* < 0.008) [29, 30]. In this study, addition of humanized anti-EGFR monoclonal antibody created conditions to reduce the intensity of induction chemotherapy; therefore, systemic toxicity and gastrointestinal reactions during induction chemotherapy were effectively controlled. Compared with DPF, the NPF regimen effectively alleviated neutropenia (*P* = 0.018) and gastrointestinal reactions (*P* = 0.032) in the induction phase, while use of EGFR monoclonal antibody in human chemotherapy did not increase mucosal-related toxicity. Toxicity control during induction therapy could also effectively improve patient tolerance during follow-up concurrent radiochemotherapy. Therefore, neutropenia, anemia, gastrointestinal reaction, oral mucositis and radiation dermatitis in the NPF group were significantly improved compared with the DPF group (*P* = 0.033, *P* = 0.049, *P* = 0.037, *P* = 0.020 and *P* = 0.035, respectively), which greatly improved the tolerance of NPF patients to whole course treatment and solved key problems that restrict the clinical application of induction chemotherapy which could create conditions for improved prognosis.

The main limitation of this study is that it only performed short-term evaluation of efficacy and adverse reactions. Therefore, long-term evaluation of the NPF regimen deserves further investigation, and would further assess the clinical significance of this regimen. In addition, the latest research shows that gemcitabine and cisplatin as induction chemotherapy added to chemoradiotherapy significantly improved LRFS and OS among patients with locoregionally advanced nasopharyngeal carcinoma [31]. Therefore, a comparison of larger sample sizes with new induced chemotherapy regimens may better confirm the role of NPF schemes.

## Conclusion

In conclusion, compared with DPF induction chemotherapy for locally advanced NPC cases receiving follow-up cisplatin and concurrent IMRT, nimotuzumab combined with PF as induction chemotherapy has better lymph node response rate, less adverse reactions, and better induction therapy and concurrent radiochemotherapy tolerance compared with the DPF regimen. Therefore, the NPF regimen may become a new choice for induction therapy of locally advanced nasopharyngeal cancer.

## Data Availability

The data set supporting the results of this article are included within the article.

## Abbreviations

CCRT: Concurrent radiotherapy and chemotherapy
CR: Complete response
CTX: Cetuximab
DC: Distance control
DFS: Disease-free survival
DMFS: Distant metastasis-free survival
DPF: Docetaxel/PF
EGFR: Epidermal growth factor receptor
IC: Induction chemotherapy
IMRT: Intensity modulated radiation therapy
LRFS: Locoregional recurrence free survival
MR: Magnetic resonance
NPC: Nasopharyngeal carcinoma
NPF: Nimotuzumab/PF
NTZ: Nimotuzumab
OS: Overall survival
PD: Progressive disease
PF: Plus 5-fluorouracil
PFS: Progression-free survival
PR: Partial response
PTV: Planning target volume
RECIST: Response Evaluation Criteria in Solid Tumors
RR: Response rate
SD: Stable disease
